# Assessing reversible and irreversible binding effects of kinase covalent inhibitors through ADP-Glo assays

**DOI:** 10.1016/j.xpro.2021.100717

**Published:** 2021-08-05

**Authors:** Martin Schröder, Apirat Chaikuad

**Affiliations:** 1 Institute of Pharmaceutical Chemistry, Goethe-University Frankfurt, 60438 Frankfurt, Germany; 2 Structural Genomics Consortium, BMLS, Goethe-University Frankfurt, 60438 Frankfurt, Germany

**Keywords:** Single-molecule Assays, High Throughput Screening, Molecular/Chemical Probes, Protein Biochemistry, Chemistry

## Abstract

Typical enzymatic inhibition assays often demonstrate improved potency for kinase covalent inhibitors compared to reversible inhibitors. This can primarily be attributed to the irreversible mode of action and could affect the evaluations of the ATP-competitive nature of covalent inhibitors, hindering optimization of these compounds. Here, we describe a version of ADP-Glo assay, in which modification of inhibitor incubation time in the presence or absence of ATP enables a quick assessment of relative reversible and irreversible effects of kinase covalent inhibitors.

For complete details on the use and execution of this protocol, please refer to Schröder et al. (2020).

## Highlights

• Simple enzymatic assays for quick assessment of kinase covalent inhibitors
• Modification of incubation time allows probing ATP-competitive nature of inhibitors
• The assays enable the evaluation of relative reversible and irreversible effects

## Graphical abstract

**Figure:**
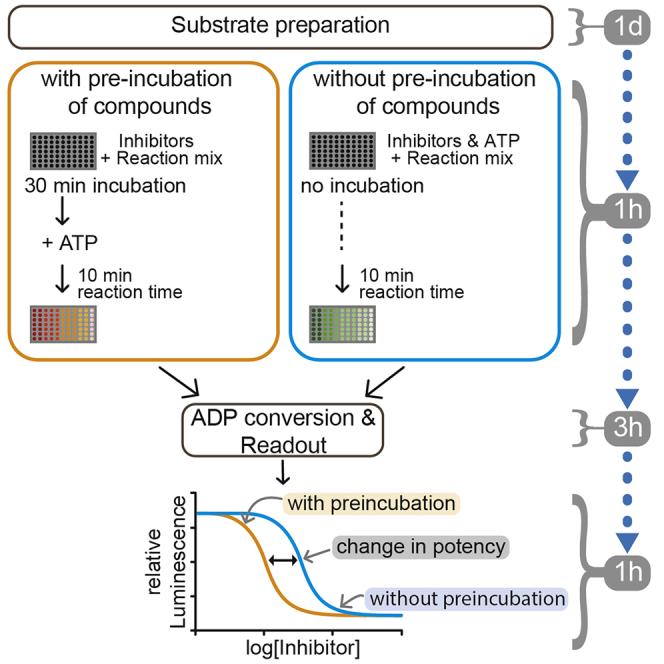

## Before you begin

The protocol described here uses ADP-Glo^TM^ to compare relative reversible and irreversible effects of covalent inhibitors, and it should be applicable for other kinases or target proteins, of which the activities can be detected by ADP-Glo^TM^ assay. While the step-by-step method should be applicable for other cases, there are a few points that may need to be considered before performing the assays. Catalytically-active kinase enzyme, a suitable substrate and optimal ATP concentration are required for ADP-Glo^TM^ kinase assays. Choice of the substrate can be peptides or proteins, which especially for the latter must not exert ATP hydrolysis activities or interact with the tested kinase inhibitors. The substrate that meets these criteria can be used directly in the assay. Nonetheless, the substrate that exerts either of these properties might also be considered, yet modification to eliminate these activities is required.

The protocol below describes the specific steps for using the protocol in a 384-well plate to evaluate the effects of covalent inhibitors on phosphorylation of JNK3 by MKK7. In this particular case, since JNK3 is also a kinase and contains a cysteine in the proximity of the ATP binding site elimination of its potential ATP hydrolysis activity and reactivity with the tested covalent inhibitors is required. One potential strategy is to use a kinase-dead form that has also its cysteine mutated. Nevertheless, we used another possible method by blocking the ATP-binding pocket of JNK3 substrate, and this is achieved by adduct formation between JNK3 and its selective covalent inhibitor Fmu-001-367 (compound 7 described by Muth et al., 2017), which could be substituted however by other alternative commercially available irreversible inhibitors, such as JNK-IN-7 or JNK-IN-8. In addition, an automated Echo® 550 acoustic liquid handler is used in this protocol. However, we have also performed this protocol with manual pipetting, in which adjusting the volume of each component of the kinase reaction is required to maintain the 5-μL volume of the reaction.

### Preparation of JNK3-Fmu-001-367 adduct

**Timing: 1 day**

**Figure 1:**
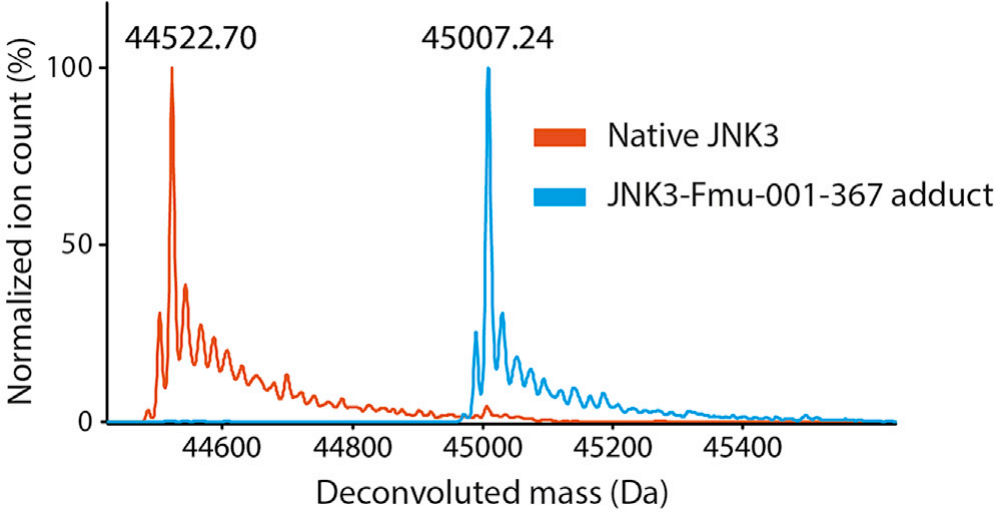
An example of the deconvoluted masses of native JNK3 and the purified JNK3-Fmu-001-367 adduct determined by mass spectrometry demonstrates the completion of the adduct formation as indicated by a mass shift and no trace of native JNK3 in the adduct sample

1. Dilute recombinant JNK3 to 20 μM at 15 mL volume using the protein storage buffer (see Material and Equipment section).

2. Add JNK-specific covalent inhibitor Fmu-001-367 to a final concentration of 30 μM, and incubate the reaction on ice for 2 h.
  ***Optional:*** The completion of the reaction can be checked by intact LC/MS.
  a. Mix 5 μL of the reaction with 95 μL of 0.1% formic acid.
  b. Inject 1 μL into Agilent 6230 TOF mass spectrometer equipped with a 300SB-C3 column (Agilent).
  c. Determine the mass of the sample by deconvoluting the measured m/z spectra using MassHunter BioConfirm software. For completed reaction, only one the adduct species with correct mass, which is the sum of the protein and inhibitor masses, should be observed (see example in Figure 1).

3. Concentrate the JNK3- Fmu-001-367 adduct to 500 μL using a 30-kDa cut off Amicon® Ultra Centrifugal concentrator and transfer the concentrated protein into a new tube. Spin down any precipitations at 15,000 × *g* for 5 min at 4°C.
4. Purify the adduct by size exclusion chromatography on Äkta liquid chromatography system (e.g., Äkta Pure) using Superdex 200 Increase 10/300 GL column pre-equilibrated with the protein storage buffer (see Material and Equipment section).
5. Concentrate the purified JNK3-Fmu-001-367 adduct (hereafter referred to as “cov-JNK3 substrate”) using a 30-kDa cut off Amicon® Ultra Centrifugal concentrator to 300 μM. This is the 10× substrate stock solution. The concentration can be measured by absorbance method at 280 nm, unless the inhibitor absorbs light at this wavelength in which case an alternative method is required (see critical note).
6. Flash freeze 10× cov-JNK3 substrate in aliquots at 50-μL volume.

**CRITICAL:** Completion of the covalent adduct formation between JNK3 and Fmu-001-367 is crucial. This is to avoid contamination of native JNK3, which can interfere the assays through non-specific interaction with the tested MKK7 inhibitors or potential ATP hydrolysis catalyzed by JNK3. The optional step in the protocol allows detection of the remaining trace of native JNK3 and is recommended but not essential. Typically, 2 h incubation of JNK3 with 1.5-fold excess of its selective covalent inhibitor should be sufficient for completion of the adduct formation (step 2). Nevertheless, prolonging incubation time to 4–6 h with a slight increase of the inhibitor to 2-fold molar excess can enable higher efficiency.

Removal of the remaining trace of Fmu-001-367, which can also interfere with the assays through non-specific interaction with MKK7, is crucial. Thus, performing size exclusion chromatography in step 3 is highly recommended. However, an alternative method such as buffer-exchange using centrifugal concentrators can also be instead employed.

In the case that other JNK3 covalent inhibitors are used, it is important to check their potential absorbance at 280 nm, which may interfere the measurement of the JNK3-inhibitor adduct concentration by UV in step 5. When the inhibitors exhibit strong absorbance property, alternative methods, such as Bradford assay, should be instead employed.

### Determination of an optimal MKK7 concentration

**Timing: 3–4 h**

7. Thaw ADP-Glo^TM^ assay reagents. Once thawed, immediately place the supplied 10 mM ultra-pure ATP solution on ice while other reagents can be kept at 22°C–25°C.

***Optional:*** we recommend that upon the first time use of the assay kit the stock of the ultra-pure ATP solution should be divided into single-use 50-μL aliquots to avoid multiple freeze/thaw cycles.

**Figure 2:**
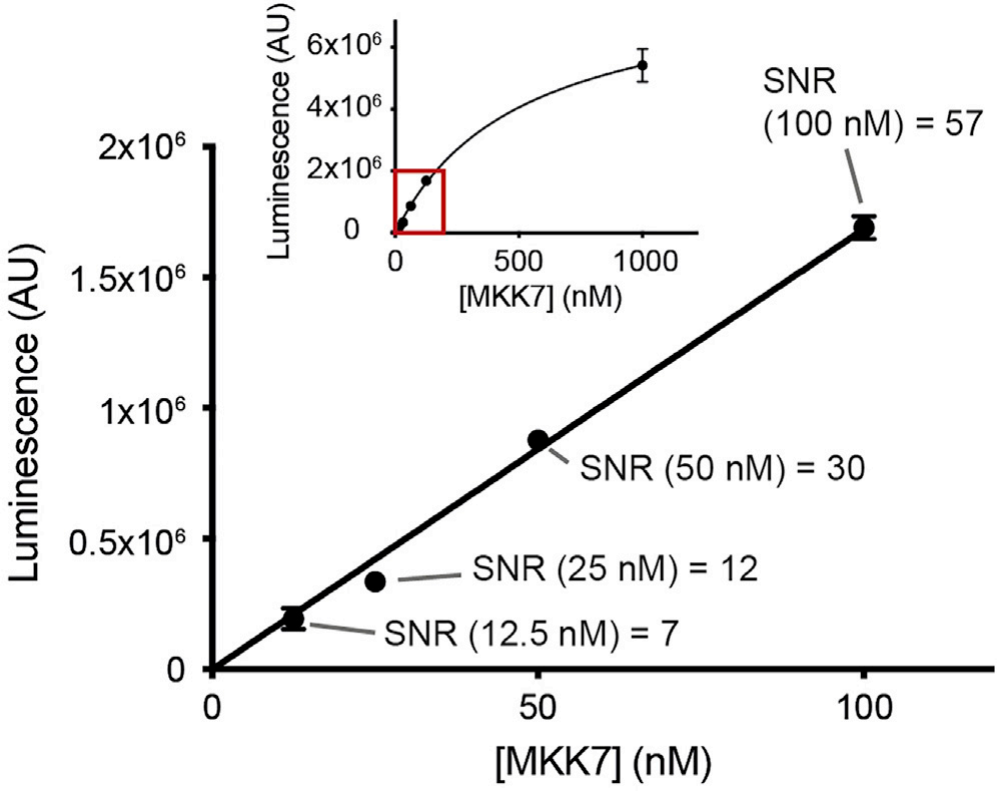
An example plot of the luminescence signals against the kinase concentrations with SNR values Shown in the main is the linear range from the full plot (red box in the inset) from which the lowest concentration of kinase with SNR of >10 (e.g., 25 nM) is considered as an optimal concentration for the later assay.

***Note:*** Once the kinase is determined to be catalytically active, DMSO at 1% final concentration might be supplemented in this protocol to measure the effect of DMSO on kinase activity, hence optimal kinase concentration, that might be observed in the later inhibition assays (see troubleshooting problem 2). This can be done by first adjusting the aliquot volume of the substrate solution in step 9 to 3.9 μL and then adding also 50 nL of 100% DMSO in step 11 by using the same procedure as described for ATP addition in this step.

8. Thaw an aliquot of 10× cov-JNK3 substrate, and quickly place the tube on ice.
9. Prepare the substrate solution by mixing 50 μL of 10× cov-JNK3 substrate with 350 μL of the reaction buffer (see Material and Equipment section). Subsequently, aliquot 3.95 μL of this substrate solution into 24 wells of a 384-well assay plate (2 sets of 12 reactions) using a multichannel pipette (electronic E1-ClipTip or equivalent).
10. Thaw MKK7 kinase and immediately prepare 10 μL of 5× kinase solution at 11 concentrations ranging from 5 nM-5 μM by half serial dilutions using the reaction buffer. Subsequently, transfer 1 μL of each kinase solution into the corresponding wells (2 sets of 11 reactions), giving thus the final kinase concentration ranging from 1–1000 nM in each set. Add 1 μL of the reaction buffer in the 12^th^ wells of both sets, which are used as a control (Control No Kinase).
11. Add ultra-pure ATP to all wells at a final concentration 0.1 mM by aliquoting 50 nL of 10 mM ultra-pure ATP stock using the plate reformat program in Echo® 550 acoustic handler (referred to the manufacture’s protocol for detailed instructions).
12. Briefly mix the kinase reaction by shaking the assay plate using a plate shaker (such as MixMate® plate shaker; ∼500 × *g*, 15 s), then incubate the reaction at 4°C for 10 min (troubleshooting 1).
13. Add 5 μL of the ADP-Glo™ reagent in all wells. Mix by shaking the assay plate briefly using a plate shaker, and subsequently incubate at 22°C–25°C for 40 min.
14. Add 10 μL of the Kinase Detection Reagent. Mix by shaking on a plate shaker, and subsequently incubate at 22°C–25°C for another 40 min.
15. Measure luminescence using PheraStar FSX plate reader (or other alternatives that can measure luminescence). We recommend either adjusting the gain values based on the intensities of the reactions with the highest kinase concentration to avoid saturation of the detector or using filters that only collect luminescence signal at certain wavelengths (referred to the manufacture’s protocol for detailed instructions).
16. Plot the measured luminescence signals against the kinase concentrations, and calculate the signal-to-noise (SNR) ratio for each kinase concentration (see Table 1 and quantification and statistical analysis). The optimal MKK7 concentration is determined by the lowest concentration that produces reliable, measurable luminescence signals (e.g., the concentration in a linear range of the plot) as well as has the SNR value of more than 10 (Figure 2).

**Table 1:** An example of the derived data from the kinase concentration determination step and the calculated SNR

Measured data	Well	0.10 μM MKK7	0.05 μM MKK7	Control No kinase
	A	1690804	877073	25256
	B	1605305	827155	32257
Calculation				
Average Control No Kinase		26786		
STDEV.S Control No Kinase		7737		
Average 0.10 μM MKK7		1648055		
STDEV.S 0.10 μM MKK7		60457		
SNR 0.10 μM MKK7		57.31		
Average 0.05 μM MKK7		852114		
STDEV.S 0.05 μM MKK7		35297		
SNR 0.05 μM MKK7		29.63

## Key resources table

**Table:** 

REAGENT or RESOURCE	SOURCE	IDENTIFIER
**Chemicals, peptides, and recombinant proteins**		

Recombinant MKK7 (full-length, S287D/T291D)	Schröder et al., 2020.	N/A
Recombinant JNK3	Schröder et al., 2020; Lange et al., 2015	N/A
Fmu-001-367	Muth et al., 2017	Compound 7
Ibrutinib	Selleckchem	S2680
OTSSP167	Selleckchem	S7159
CPT1-70-1	Tan et al., 2017 and patent WO2016130920	WO2016130920: Compound 44

**Critical commercial assays**		

ADP-Glo™	Promega	V6930

**Software and algorithms**		

BMG LABTECH microplate reader software	BMG Labtech	https://www.bmglabtech.com/microplate-reader-software/
Microsoft Excel	Microsoft	n/a
Prism	GraphPad	https://www.graphpad.com/scientific-software/prism/
MassHunter BioConfirm	Agilent	https://www.agilent.com/en/product/software-informatics/mass-spectrometry-software/data-analysis/bioconfirm-software

**Other**		

384-Well assay plate (PS, flat bottom, low volume, white)	Greiner Bio-One	784075
Amicon® Ultra Centrifugal Concentrator	Merck Millipore	Z740204
ÄKTA Pure	GE Healthcare	N/A
Superdex S200 Increase 10/300 GL	GE Healthcare	28990944
Echo® 550 Acoustic Liquid Handler	Labcyte	N/A
Echo® source plate	Labcyte	P-05525
MixMate® plate shaker	Eppendorf	5353000510
E1-ClipTip Electronic Multichannel Pipette	Thermo Fisher Scientific	4672050BT
Agilent 6230 TOF LC/MS	Agilent	6230
Agilent 300SB-C3	Agilent	820950-924

## Materials and equipment

**Table:** 

Protein storage buffer	Final concentration (mM)	Amount
Sterilize by filtration and store at 4°C		
NaCl (5 M)	300	40 mL
HEPES pH 7.5 (1 M)	30	20 mL
TCEP pH 7.0 (0.5 M)	0.5	1 mL
Glycerol (100%)	5%	50 mL
ddH_2_O	n/a	889 mL
**Total**		**1000 mL**

**Table:** 

Reaction buffer	Final concentration (mM)	Amount
Sterilize by filtration and store at 4°C		
MgCl_2_ (1 M)	20	20 mL
Tris pH 7.5 (1 M)	40	40 mL
ddH_2_O	n/a	940 mL
**Total**		**1000 mL**

**Table:** 

Kinase reaction mix (for reaction performed with Echo® 550 acoustic handler)	Final concentration (μM)	Amount
Sterilize by filtration and store at 4°C		
MKK7 (10X stock, 250 nM)	0.025	500 nL
Cov-JNK3 (10X stock, 300 μM)	30	500 nL
10 mM ultra-pure ATP	100	50 nL
Inhibitor/intermediate inhibitor dilutions	varying	50 nL
Reaction buffer	1×	3900 nL
**Total**		**5000 nL**

**Table:** 

Kinase reaction mix (for reactions performed with manual pipetting)	Final concentration (μM)	Amount
MKK7 (10X stock, 250 nM)	0.025	500 nL
Cov-JNK3 (10X stock, 300 μM)	30	500 nL
0.5 mM ultra-pure ATP	100	1000 nL
Inhibitor dilution series (5×)	varying	1000 nL
Reaction buffer	1×	3000 nL
**Total**		**5000 nL**

***Note:*** The final kinase reaction mix contains 31.6 mM Tris pH 7.5, 60 mM NaCl, 15.8 mM MgCl_2_, 1% glycerol, 0.1 mM TCEP, 25 nM MKK7, 30 μM cov-JNK3 substrate and 100 μM ATP. The protocol described here uses Echo® 550 liquid handler. However, when performing with manual pipetting, adjusting pipetting volume of each component of the kinase reaction is required with an example provided in the above table. We had performed the following protocol using both pipetting methods, and observed similar assay quality with an example shown in Figure 3.

**Figure 3:**
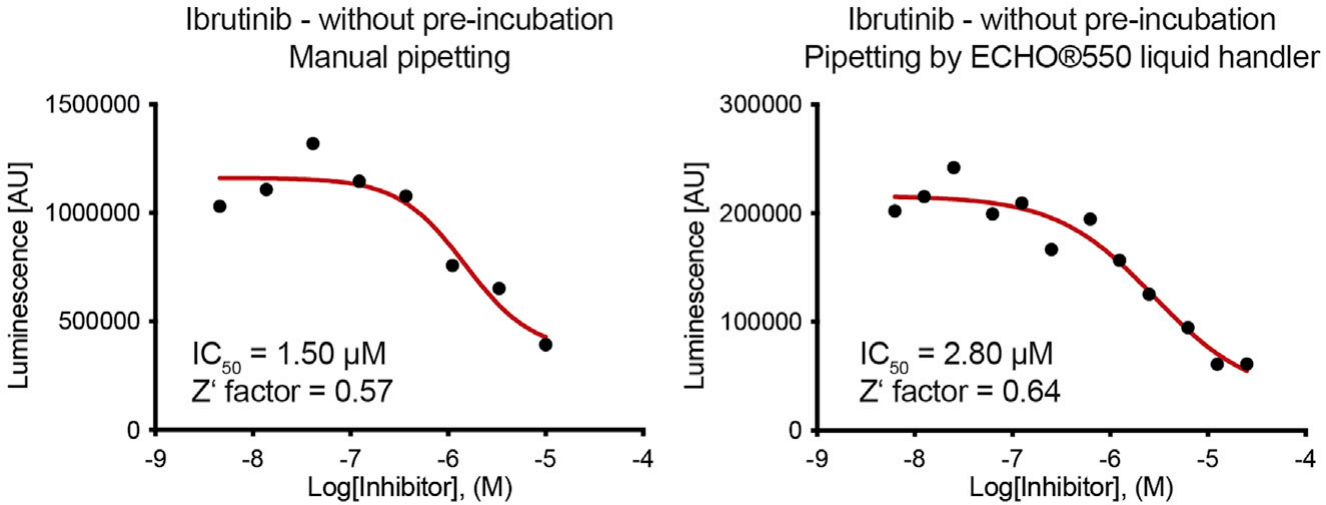
An example of the results obtained when performing the protocol using manual pipetting (left) and ECHO®550 liquid handler (right) Note the comparable Z′ factors indicating similar assay quality for both methods.

## Step-by-step method details

### IC_50_ determination using ADP-Glo^TM^ assays with a pre-incubation step

**Timing: 3–4 h**

**Figure 4:**
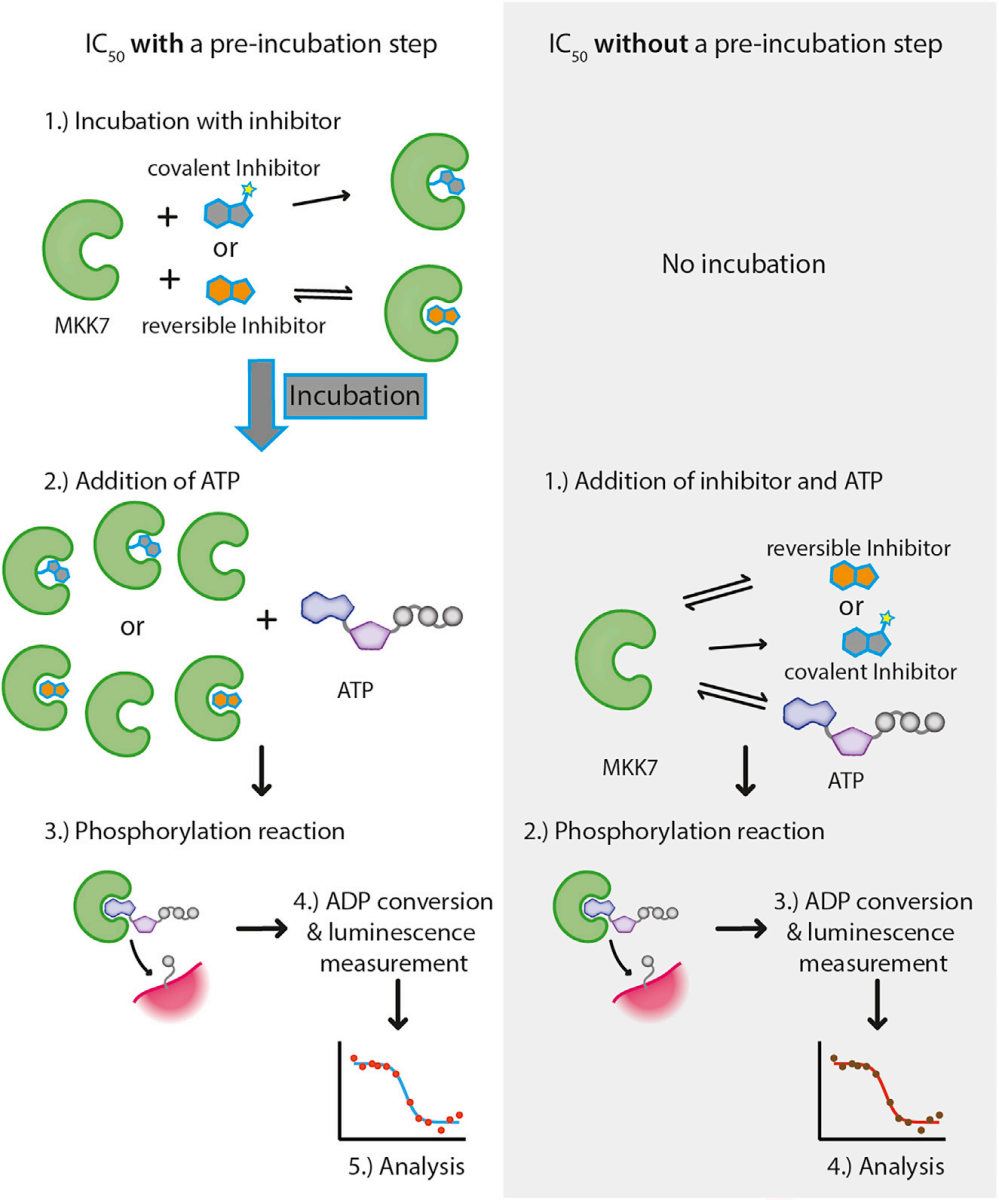
Schematic illustration of the protocol workflow

**Figure 5:**
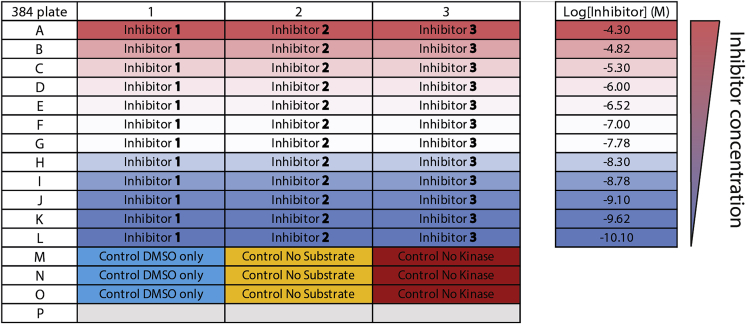
The experimental plate layout for the 384-well assay plate used in the described protocol for assessing inhibitory potencies of three inhibitors See also Data S1 for an example input for Echo® dose response pipetting, related to Step 5.

This part of the protocol describes a variation of the ADP-Glo^TM^ assay that includes a pre-incubation step between the tested inhibitors and MKK7 prior to an addition of ATP (Figure 4). In this case, the inhibitors do not compete with ATP for their interactions with the kinase; hence for covalent inhibitors more pre-formed adduct formation and less available active kinase for subsequent JNK3 phosphorylation. Thus, apparent greater inhibitory potencies would be expected. The protocol below describes an experiment testing three inhibitors using fixed concentrations of 25 nM for MKK7 kinase, 30 μM for cov-JNK3 substrate and 100 μM for ATP with the layout of the assay plate shown in Figure 5.

**Figure 6:**
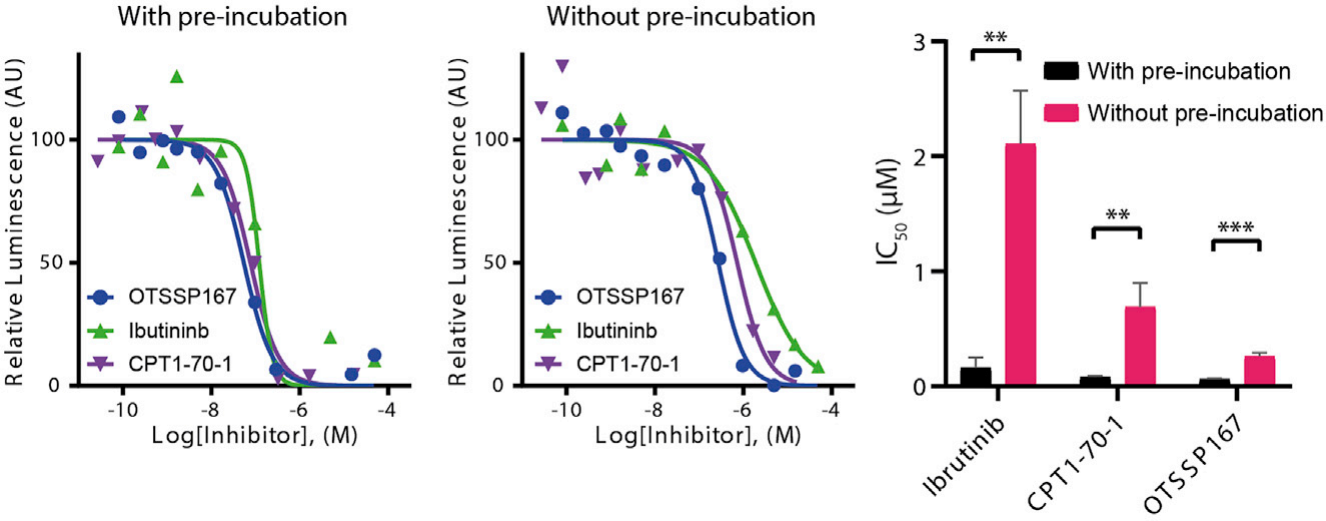
Example of the results obtained from using the protocol for assessing three MKK7 inhibitors Shown on the left and middle are the dose-response curves obtained from the experiments with and without kinase-inhibitor pre-incubation, respectively. The average IC_50_s with standard deviation from triplicates are shown on the right bar chart, depicting the shifts of potencies between two experiments typically expected for covalent inhibitors such as ibrutinib and CPT1-70-1 with the degree of changes varied depending on their ATP-competitive nature (∗∗ and ∗∗∗ indicates Student’s t-test P values of <0.01 and <0.001, respectively). These data have also been described in Schröder et al., 2020.

**Figure 7:**
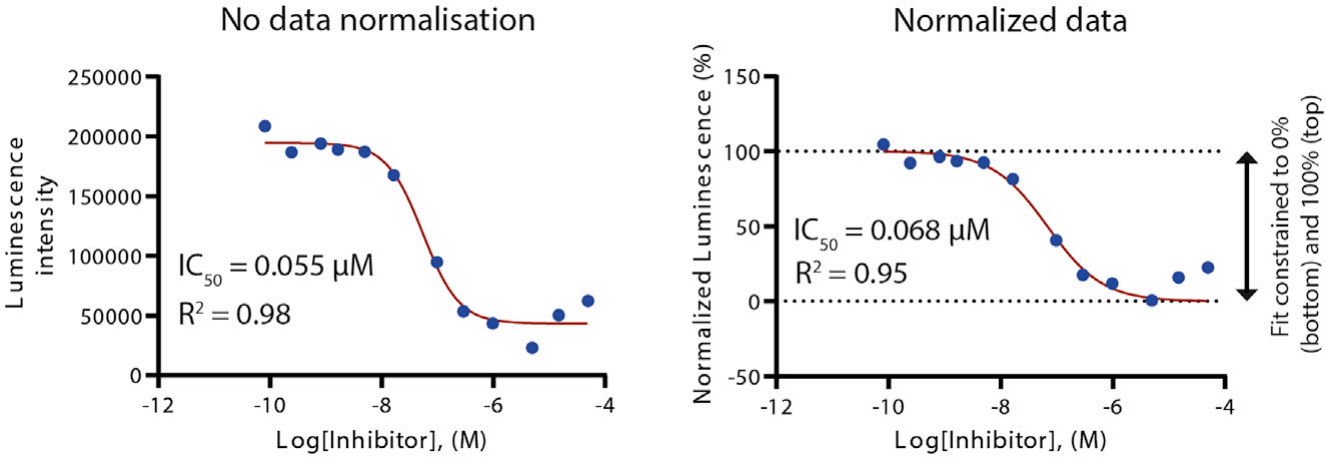
An example of dose-response fitting using either raw intensities (left) or normalized intensities (right) for IC_50_ determination Slight changes in the IC_50_ values might be expected due to the setting of minimum and maximum values at 0% and 100%, respectively, for the fitting of the normalized intensities, but both IC_50_ values should not be highly deviated for high accuracy assays determined by high Z′ factor.

1. Thaw ADP-Glo^TM^ assay reagents. Place 10 mM ultra-pure ATP immediately on ice, while the other reagents can be kept at 22°C–25°C.
2. Quick thaw MKK7 kinase and 10× cov-JNK3 substrate and spin both using benchtop centrifuge at high speed at 4°C for 10 min. Transfer clear supernatant into a new tube and keep on ice.
3. Prepare 75 μL of 10× MKK7 kinase solution by diluting MKK7 to 250 nM in protein storage buffer. Keep the solution on ice until further use.
4. Prepare the following mixtures and keep them on ice until further use.
  a. Mixture 1: one part of 10× MKK7 kinase solution, one part of 10× cov-JNK3 substrate and eight part of the reaction buffer. For example, 500 μL of Mixture 1 contains 50 μL of 10× MKK7 kinase solution, 50 μL of 10× cov-JNK3 substrate and 450 μL of the reaction buffer. This 500-μL Mixture 1 is adequate for approximately 100 reactions and will be used for the reactions containing inhibitors and the “Control DMSO only” wells (see Figure 5).
  b. Mixture 2: one part of protein storage buffer, one part of 10× cov-JNK3 substrate and eight part of the reaction buffer. For example, 50 μL of Mixture 2 is made from 5 μL of protein storage buffer, 5 μL of 10× cov-JNK3 substrate and 45 μL of the reaction buffer. This 50-μL Mixture 2 is adequate for 10 reactions and is for the “Control No kinase” wells (see Figure 5).
  c. Mixture 3: one part of 10× MKK7 kinase solution, one part of protein storage buffer and eight part of the reaction buffer. For example, 50 μL of Mixture 3 is made from 5 μL of 10× MKK7 kinase solution, 5 μL of protein storage buffer and 45 μL of reaction buffer. This 50-μL Mixture 3 is adequate for 10 reactions and is for the “Control No substrate” wells (see Figure 5)
5. Prepare a serial dilution of inhibitors in a 384-well assay plate using the “Dose response” setting in Echo®550 acoustic liquid handler according to the manufacture’s manual. For this experiment, the dilution series of three inhibitors at 11 concentrations is prepared by the following steps (see also Figure 5 and Data S1 for an example input for Echo® dose response pipetting):
  a. Prepare 5 mM inhibitor stock solutions in DMSO and transfer 25 μL of each into three different wells of an Echo® source plate. Fill the 4^th^ well with 30 μL DMSO.
  b. Use the “Dose response” program in Echo® 550 liquid handler with a DMSO backfill option to aliquot the correct volumes of the inhibitors and DMSO into each reaction wells in the assay plate according to the plate layout in Figure 5. Note that two intermediate compound dilutions will be created by the ECHO® dose response program (see Data S1 for an example input for Echo® dose response pipetting). 50 nL of DMSO should be transferred to each control well to ensure homogeneous assay conditions. The total transfer volume of 50 nL of either inhibitor alone, inhibitor plus DMSO or DMSO alone is fixed to ensure constant 1% final DMSO concentration (see Data S1 for an example input for Echo® dose response pipetting).
6. Immediately add 4.9 μL of the mixtures from step 4 into the corresponding wells of the assay plate using a multichannel pipette as following (see Figure 5 for plate layout):
  a. Mixture 2 for the three wells designated as “Control No kinase”.
  b. Mixture 3 for the three wells designated as “Control No substrate”.
  c. Mixture 1 for all wells with pre-added inhibitors and the three “Control DMSO only” wells.
7. Briefly mix the kinase reactions by shaking the assay plate with a plate shaker (e.g., MixMate® plate shaker; 500 × *g*, 15 s), then incubate the reactions at 4°C for 30 min.
8. Add 50 nL of 10 mM ultra-pure ATP in all reactions to give 0.1 mM final concentration using the “Plate reformat” program in Echo®550 acoustic handler (refer to the manufacture’s manual).
9. Briefly mix by shaking the assay plate using a plate shaker, then incubate the reactions at 4°C for 10 min (see troubleshooting 1).
10. Add 5 μL of the ADP-Glo^TM^ Reagent in all wells using a multichannel pipette. Mix by shaking with a plate shaker, and subsequently incubate at 22°C–25°C for 40 min.
11. Add 10 μL of the Kinase Detection Reagent using a multichannel pipette. Mix by shaking with a plate shaker, and subsequently incubate at 22°C–25°C for another 40 min.
12. Measure luminescence using PheraStar FSX plate reader (or other alternatives that can measure luminescence). For high accuracy, we recommend either adjusting the gain setting of the plate reader based on the intensity of the “Control No kinase” wells to avoid saturation of the detector or using filters that only collect luminescence signal at certain wavelengths (referred to the manufacture’s protocol for detailed instructions).
13. Determine the IC_50_ values (see Figures 6 and 7 and Tables 2 and 3):
  a. Plot the measured intensities (Y axis) against the inhibitor concentrations in logarithmic scale (X axis). If necessary, use the intensity values that are normalized against the “DMSO only” and “No kinase” controls (see quantification and statistical analysis)
  b. Use dose-response fitting, e.g., “log (inhibitor) vs. response-Variable slope (four parameter)” fitting in GraphPad Prism software, to calculate IC_50_ values for the inhibitors.
  c. For validation of the accuracy of the assays, we recommend calculating also the Z′ values (see quantification and statistical analysis).

**Table 2:** An example of the derived data from the protocol and the calculated Z′ factor

Measured data	Well	1	2	3
	…	…	….	…
	L	…	…	…
Controls	M	202290	30062	23634
Controls	N	212221	37664	22385
Controls	O	186130	22081	19947
		Control DMSO only	Control No Substrate	Control No Kinase
Calculation				
Average Control DMSO only		200214		
STDEV.S Control DMSO only		13169		
Average Control No Kinase		21989		
STDEV.S Control No Kinase		1875		
Z′ factor	Z′ factor	0.75

**Table 3:** An example of normalization for the derived data from the protocol

			Ibrutinib	CPT1-70-1	OTSSP167
[Inhibitor] (M)	log[Inhibitor]	Well	1	2	3
4.95E-05	-4.31	A	52382	23634	62181
1.49E-05	-4.83	B	18884	13859	50306
4.95E-06	-5.31	C	64212	22549	22921
9.71E-07	-6.01	D	22472	20754	43374
2.91E-07	-6.54	E	49318	93951	53403
9.71E-08	-7.01	F	121319	127811	94669
1.65E-08	-7.78	G	157972	159768	167511
4.95E-09	-8.31	H	138435	176579	186941
1.65E-09	-8.78	I	195602	171671	188762
8.08E-10	-9.09	J	152383	188847	193825
2.43E-10	-9.62	K	176476	170474	186492
8.08E-11	-10.09	L	159956	157801	208349
	Controls	M	202290	30062	23634
	Controls	N	212221	37664	22385
	Controls	O	186130	22081	19947
			Control DMSO only	Control No Substrate	Control No Kinase
			Ibrutinib	CPT1-70-01	OTSSP167
		log[Inhibitor]	Normalized Intensity (%)		
		-4.31	17.05	0.92	22.55
		-4.83	-1.74	-4.56	15.89
		-5.31	23.69	0.31	0.52
		-6.01	0.27	-0.69	12.00
		-6.54	15.33	40.38	17.63
		-7.01	55.73	59.38	40.78
		-7.78	76.30	77.31	81.65
		-8.31	65.34	86.74	92.55
		-8.78	97.41	83.99	93.57
		-9.09	73.16	93.62	96.42
		-9.62	86.68	83.31	92.30
		-10.09	77.41	76.20	104.56

**CRITICAL:** Due to the reactivity nature of covalent inhibitors, the addition of Mixture 1, 2 and 3 in step 6 must be completed as quickly as possible to ensure consistency regarding the kinase-inhibitor incubation time across the assay plate. We suggest that the solution transfer in this step should be first completed for all control wells for high accuracy of background measurement. In addition, it is important to maintain 1% DMSO in all reactions.

### IC_50_ determination using ADP-Glo assays without a pre-incubation step

**Timing: 3–4 h**

This part of the protocol describes the other variation of the ADP-Glo assay, in which no kinase-inhibitor pre-incubation step is included and both covalent inhibitors and ATP are added into the reaction at the same time (Figure 4). In this scenario, the inhibitors compete with the physiological ATP substrate for their interactions with the kinase. Thus, this allows probing the ATP-competitive reversible binding of the covalent inhibitors. Lower inhibitory potencies compared to that obtained from the earlier assays with a pre-incubation step would be an expected outcome, especially for covalent inhibitors with weak ATP competitions of which the inhibitory efficacy is driven primarily by covalent mode of action. The protocol below should be performed in parallel with the above assays with a pre-incubation step, preferably on the same day with the same stock solutions and the same experimental designs.

14. Thaw ADP-Glo^TM^ assay reagents, MKK7 and 10× cov-JNK3 substrate as described in step 1 and 2.
15. Prepare 75 μL of 10× MKK7 kinase solution by diluting MKK7 to 250 nM in protein storage buffer. Keep the solution on ice until further use (same as step 3).
16. Prepare Mixture 1, 2 and 3 as described in step 4 and keep them on ice until further use.
17. Prepare a serial dilution of inhibitors in a 384-well assay as described in step 5.
18. Add 50 nL of 10 mM ultra-pure ATP in all reactions to give 0.1 mM final concentration using the “Plate reformat” program in Echo®550 acoustic handler as described in step 8.
19. Immediately add 4.9 μL of the mixtures from step 16 into the corresponding wells of the assay plate using a multichannel pipette as following:
  a. Mixture 2 for the three wells designated as “Control No kinase”.
  b. Mixture 3 for the three wells designated as “Control No substrate”.
  c. Mixture 1 for all wells with pre-added inhibitors and the three “Control DMSO only” wells.
20. Briefly mix the kinase reactions by shaking the assay plate with a plate shaker, then incubate the reaction at 4°C for 10 min.
21. Add 5 μL of the ADP-Glo^TM^ Reagent into all wells using a multichannel pipette. Mix by shaking the assay plate on a plate shaker and subsequently incubate at 22°C–25°C for 40 min.
22. Add 10 μL of the Kinase Detection Reagent using a multi-channel pipette. Mix by shaking on a plate shaker and subsequently incubate at 22°C–25°C for another 40 min.
23. Measure luminescence using PheraStar FSX plate reader as described in step 12.
24. Determine the IC_50_ values using the same procedure as described in step 13.

**CRITICAL:** We highly recommend that both variants of the assays, with and without inhibitor pre-incubation, should be performed in parallel preferably on the same day using the same stock solutions of MKK7, cov-JNK3 substrate and ATP. Similar to the experiment with a pre-incubation step, the addition of Mixture 1, 2 and 3 in step 19 must be completed as quickly as possible to avoid variation regarding kinase-inhibitor-ATP incubation time across the assay plate. We suggest that the solution transfer in this step should be first completed for all control wells for high accuracy of background measurement. In addition, 1% DMSO should be kept constant in all reactions.

## Expected outcomes

An example of the expected results when using the protocol for testing three MKK7 inhibitors, including covalent inhibitor ibrutinib and CPT1-70-1 and reversible OTSSP167, is shown in Figure 6 with two sets of complete dose-response inhibition curves and IC_50_ values. While the inhibitory potencies obtained from the assays with a pre-incubation of the kinase and inhibitors are comparable, different efficacies among these inhibitors are apparent when no pre-incubation step is included. For covalent inhibitors with weak ATP competitions due to suboptimal reversible binding, such as ibrutinib, a significant shift of inhibitory potencies between two assay variants is expected. In contrast, the changes of IC_50_s are less pronounced for covalent inhibitor CPT1-70-1 and non-covalent inhibitor OTSSP167, likely due to their greater ATP-competitive nature. Overall, this simplified ADP-Glo^TM^ enzymatic assays enable quick relative assessment of reversible and irreversible efficacies, allowing a better comparison of kinase covalent inhibitors. Further examples of the results from this protocol are demonstrated in Schröder et al. (Schröder et al., 2020) in which the assays have been used successfully for comparison of diverse MKK7 inhibitors*.*

## Quantification and statistical analysis

The signal-to-noise ratio (SNR) used for determining the optimal kinase concentration is calculated by the following equation:

$$SNR=\frac{\mu P}{\mu N}$$

where μP is the average luminescence intensity of the wells containing different kinase concentrations and μN is the average luminescence intensity of the “Control No Kinase” reaction. Table 1 demonstrates an example of the SNR calculation for different kinase concentration in the optimization process (see section ‘Determination of an optimal MKK7 concentration’). An optimal kinase concentration is the lowest concentration with SNR of >10 (Figure 2).

The Z′ factor is calculated based on the luminescence intensities of the control wells using the following equation (Zhang, Chung, & Oldenburg, 1999):

$$Z^{\prime }=1-\frac{3\left(\sigma_{P}+\sigma_{N}\right)}{\left|\mu_{P}-\;\mu_{N}\right|}$$

where μP is an average luminescence intensity of the “Control DMSO only” wells, μN is an average intensity of the control with no MKK7 kinase (“Control No Kinase”) and σP and σN are the standard deviations for these two controls, respectively. Table 2 shows an example of the Z′ factor calculated from this data set with a value of 0.75 indicating a good assay quality. Generally, robust and high accuracy assays should have Z′ factor of >0.5 (Zhang et al., 1999).

The measured intensities can be normalized against the intensities of the “Control DMSO only” and “Control No kinase” using the following equation:

$$Normalized\;Intensity\left(x\right)=100\%\times \frac{\left(measured\;\mathrm{int}ensity\left(x\right)-\mathrm{int}ensity\;of\;"Control\;No\;Kinase"\right)}{\mathrm{int}ensity\;of\;"Control\;\text{DMSO}\;only"-\mathrm{int}ensity\;of\;"Control\;No\;Kinase"}$$

where ‘measured intensity (x)’ is the luminescence intensity of the reaction *x* and the ‘intensity of “Control No-kinase”’ and ‘intensity of “Control DMSO-only”’ are an average luminescence intensity of “Control No Kinases” and “Control DMSO only”, respectively. When using the normalized data, the minimum and maximum values for the dose-response fitting should be set to 0% and 100%, respectively. Table 3 demonstrates an example of data normalization, which is used in the dose-response fitting and IC_50_ calculation shown in Figure 7.

## Limitations

**Figure 8:**
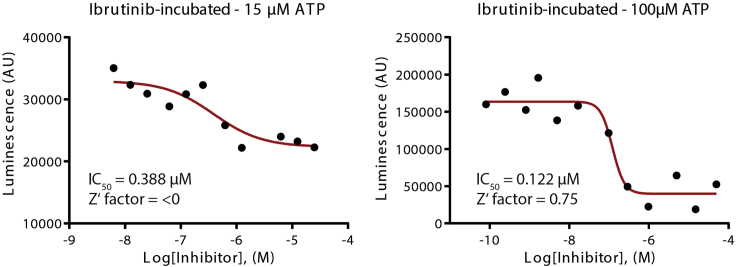
An example of the dose response curves obtained from two assays with different ATP concentrations demonstrates low assay quality when low ATP concentration (e.g., 15 μM) is used

The accuracy of this protocol may be reduced for covalent inhibitors with exceptionally high potencies, especially when the IC_50_ values are much lower than half of the used kinase concentration. This might be counteracted by increasing the ATP concentration to 0.5–1 mM or lower the kinase concentration, providing that luminescence intensities remain detectable and can be accurately measured. Nonetheless, another orthogonal assay should be considered for comparison. We recommend also using the ATP concentration of at least 0.1 mM for better distinguishable IC_50_’s between the two assay variants as low ATP concentration (e.g., 0.015 mM) might impair the assay quality (see Figure 8). In addition, for covalent inhibitors that react with cysteine a caution must be made regarding the presence of reducing agents. We have performed this protocol in the presence of TCEP at 0.1 mM without observing significant interference. Moreover, the speed of solution transfers at various steps can significantly alter the outcome as described in critical notes. When available, we thus suggest using a multichannel pipette together with an automated liquid handler as outlined in the protocol. Nevertheless, we have performed this protocol successfully with manual pipetting alone. However, adjustment of the volumes of the components in the kinase reaction as well as an optimal solution transfer speed may be required.

## Troubleshooting

### Problem 1

Low luminescence intensity.

This problem may arise when there is little ATP-to-ADP conversion.

### Potential solution

Check the activity of MKK7 kinase. Several factors can affect this, essentially protein stability. We recommend using a freshly-thawed stock of the kinase, and if necessary performing the phosphorylation reactions at higher temperature (e.g., 25 or 37°C). Furthermore, increasing incubation time in step 9 and 20 could also be considered. Alternatively, this problem may arise from degraded ATP through hydrolysis (see problem 4). Making small single-use aliquots of ATP is therefore highly recommended as multiple freeze/thaw cycles can lead to high degree of ATP hydrolysis. If problem persists, fresh stock of ATP should be considered.

### Problem 2

Unusually high background intensity.

High level of background luminescence is observed even in the control well without kinase, which thus generally reduces the assay window.

### Potential solution

An increase in background signals can occur over time, which could arise through the use of old ATP stock or the stock that is gone through multiple freeze/thaw cycles, hence a potential accumulation of ADP though autohydrolysis. When this is the case, high intensity should also be observed for the control wells (see step 6 and 19). Testing this by performing the assays for the controls using the fresh and old ATP stock in parallel for comparison. If high background intensity is observed for the old ATP solution but not the fresh stock, it is likely that high ADP level accumulated through autohydrolysis is the cause. In this case, the problem can be solved by using a fresh stock of ATP in step 8 and 18. We recommend also making small single-use aliquots of ATP to avoid multiple freeze/thaw cycles.

Another potential cause could be ATP hydrolysis activity of the other substances in the assay, such as substrate especially when another kinase is used (e.g., JNK3 in this protocol). In this case, high background intensity would be observed for the ‘Control No kinase’ and ‘Control DMSO only’ but not the ‘Control No substrate’ (see step 6 and 19). The change of substrate is therefore recommended (see ‘before you begin’ section).

### Problem 3

Unusual lower kinase activity in the inhibition assays than the assay optimization.

The luminescence intensities of all reactions, essentially the controls containing DMSO only, are significantly lower than that observed in the optimization step (determination of optimal MKK7 concentration).

### Potential solution

DMSO may affect kinase activity. Where possible, the stock concentrations of inhibitors used in step 5 and 17 can be increased to enable less transfer volume, hence lowering DMSO concentration. Alternatively, perform the assay optimization in the presence of DMSO.

### Problem 4

Inhibitor potency exceeds the assay limit.

The IC_50_ values are lower than half of the kinase concentration (e.g., for this protocol IC_50_ <12.5 nM when MKK7 kinase is used at 25 nM), leading to inaccurate measured inhibitory potencies.

### Potential solution

Increase the ATP concentration in step 8 and 18, which can generally reduce the inhibitor potencies. Alternatively, use lower kinase concentration, providing that luminescence intensities remain detectable and assay quality is maintained (see the Z′ factor). In addition, supplementation of a non-covalent inhibitor at a fixed concentration (e.g., as a component in the reaction buffer, see material and equipment section) could present a possible solution as this would provide an additional competitor that can lower apparent potencies of highly potent covalent inhibitors.

### Problem 5

Unchanged inhibitory potencies between the two assay variants.

The shifts in the potencies of a covalent inhibitor between two assay variants are insignificant or less than that observed for reversible inhibitors.

### Potential solution

This problem may arise from too slow speed in solution transfer in step 6 and 19, leading to an increase or reduction of the kinase-inhibitor incubation time. Optimizing pipetting speed in these steps is therefore recommended (e.g., practicing transferring water in the relevant steps in the protocol). In addition, this could be due to chemically-degraded irreversible inhibitors that loss their covalent binding abilities. In this case, we suggest testing reactivity of the covalent inhibitors, such as assessing the adduct formation using mass spectrometry. We recommend also using a freshly prepared stock of covalent inhibitors for comparison. Furthermore, increasing the ATP concentration (e.g., 1 mM) to provide greater competition should be considered. If the problem persists, it is likely that the assay reaches its limitation, which could be due to the intrinsic natures of the inhibitors such as strong ATP competition and high efficacies of reversible binding. For this case, it might be worth considering using a non-covalent inhibitor as an additional competitor as described in the potential solution of Problem 4.

## Resource availability

### Lead contact

Further information and requests for resources and reagents should be directed to and will be fulfilled by the lead contact, Apirat Chaikuad, chaikuad@pharmchem.uni-frankfurt.de.

### Materials availability

This study did not generate new unique reagents.

### Data and code availability

This study did not generate datasets.

## Supplemental information

Data S1. An example input for Echo® dose response pipetting, related to step 5 of the protocol
